# Spoilt for Choice in Undergraduate Medical Admission: Selection of Confident and Considerate Students Using Professional Prequalification and Situational Judgement Test

**DOI:** 10.5334/pme.1571

**Published:** 2025-06-23

**Authors:** Ina Mielke, Wolfgang Hampe, Maren Ehrhardt, Mirjana Knorr

**Affiliations:** 1 Department of Biochemistry and Molecular Cell Biology, University Medical Center Hamburg-Eppendorf, Germany; 2 Department of General Practice/Primary Care, University Medical Center Hamburg-Eppendorf, Germany

## Abstract

**Introduction:**

When considering personal characteristics in undergraduate medical admission, professional prequalification (i.e., previous work experience in the medical field) and performance on situational judgement tests (SJTs) are prominent criteria. Despite evidence on the ability of professional prequalification and SJT performance to predict general interpersonal skills, their benefit for students’ concrete behavior in everyday situations remains unclear.

**Methods:**

With the present study, we examined how undergraduate medical students with prior professional qualification and students with better SJT performance act in actual social situations by using a behavior-focused observer approach. Supervisors rated the students after a mandatory one-week practical training in general medicine on three distinguishable interpersonal dimensions, namely agency (i.e., assured-dominant), communion (i.e., warm-agreeable), and emotional stability (i.e., calm and stable). Prior to admission, participants (*N* = 210) completed a traditional SJT (i.e., HAM-SJT), a natural science test (i.e., HAM-Nat), and responded to sociodemographic questions.

**Results:**

Controlling for cognitive admission criteria, age, and gender, students with prior professional qualifications were rated as more assured-dominant (*β* = 0.21; 95% CI [0.04; 0.39], *p* = 0.019), whereas students with better HAM-SJT performance were rated as more warm-agreeable (*β* = 0.17, 95% CI [0.02; 0.31], *p* = 0.024).

**Discussion:**

The results value both admission criteria as complements to cognitive criteria in undergraduate selection but emphasize their differentiated focus on students’ interpersonal behavior. Medical schools are invited to review their admission criteria based on the present findings and on the question of required and learnable skills.

## 1. Introduction

Medical schools are increasingly interested in selecting undergraduate students who not only have the cognitive ability for successful graduation, but possess other relevant personal characteristics (e.g., social skills) to become good physicians [1,2,3,4]. The definition of relevant personal characteristics and the repertoire of related assessment methods vary between countries and medical schools [4,5]. In early stages of admission with high numbers of applicants, two internationally recognized criteria for screening personal characteristics are professional prequalification and the use of situational judgement tests (SJTs) (e.g., 6). Although both criteria are popular, there remains uncertainty about their validity, that is, which favorable personal characteristics they actually assess. With the present study, we provide another argument for the validity of professional prequalification and SJT performance in undergraduate medical admission by examining how both criteria are related to supervisor ratings of students’ interpersonal behavior.

Professional prequalification can be broadly defined as all types of an individual’s work experience related to medical training prior to studying medicine. Formal operationalization typically considers long-term activities with practical components in a healthcare related profession, such as volunteering, community service, or completion of a vocational training or degree (i.e., in nursing or as a paramedic) [7]. In addition, acquired experiences and knowledge may indirectly influence performance on other selection criteria such as in-person interviews [8]. The theoretical rationale for the use of professional prequalification in selection is based on the assumption that experienced applicants have already developed important personal and social skills including resilience [9] and professionalism [10], and that these skills facilitate learning processes during studies which, consequently, lead to study success [9]. However, evidence to support this idea is generally scarce and results are mixed. While medical applicants with previous vocational training performed better in interviews assessing personal and social skills [11,12], no association was found between medical students’ previous work experience and ratings of their professionalism after a one-year internship [13] or communication performance in an objective structured clinical examination (OSCE) [14]. As professional prequalification does apparently not improve performance in every interpersonal task, a deeper analysis of experienced students’ specific behavioral tendencies would improve the understanding of its potential benefits for social interactions in the workplace.

In contrast to professional prequalification, the prediction of interpersonal skills by SJTs assessing personal characteristics in undergraduate selection is well documented throughout medical education [15,16,17]. The basic idea of SJTs is that applicants’ evaluations of responses to critical interpersonal situations reveal something about their behavioral preferences which is consistently reflected in actual future behavior in similar situations [18]. The development of SJTs is usually based on the nature of everyday study or work situations (traditional approach) [19], resulting in multidimensional items and an aggregated score that reflects general effective behavior [20,21]. An example of a traditionally developed SJT is the HAM-SJT which was recently introduced in Germany to screen for a mixture of personal and social skills in medical applicants [22,23]. Although the use of such traditional SJTs for admission can be justified by their prediction of behavior-based performance (e.g., in multiple mini interviews [MMI] or OSCEs) [24], the understanding of what they actually measure should be of great interest when making important admission decisions [19]. Consistent with our outline for professional prequalification, we suggest that examining how SJTs predict behavior in different interpersonal dimensions, rather than how they predict other broad composite scores from a high-stakes context, will promote the understanding of their construct validity.

### 1.1. Present Research

The general aim of the present study is to understand how professional prequalification and SJT performance are related to ratings of interpersonal behavior in real-life work situations. These insights are crucial to understanding how both selection criteria can predict students’ actual behavior when interacting with patients, relatives, or colleagues.

Interpersonal behavior is rated by multiple supervisors after a one-week practical training with respect to three distinct and observable dimensions: agency, communion, and emotional stability [25]. Agency and communion denote two fundamental and well-established factors in interpersonal theory [26] that highlight an individual’s motive to go ahead and achieve their own goals or to get along and establish relationships in social situations [27]. Both factors subsume different behaviors, and we focus on assured-dominant behavior (agency) [28] and warm-agreeable behavior (communion), which we consider important for medical students [29,30]. Emotional stability describes a tendency to stay calm and stable and is associated with higher levels of resilience [31].

As evidence for relations between concrete behavioral tendencies and professional qualification or SJT performance is scarce, we examine this as an open research question.^1^ We also examine the incremental validity in predicting interpersonal behavior beyond established cognitive selection criteria (i.e., grade point average [GPA] and a natural science test [HAM-Nat]) [32] and demographics (i.e., gender and age).^2^

## 2. Methods

### 2.1. Participants and Procedures

This was a longitudinal prospective study that followed participants from admission to completion of a practical training after the first year of study. Participants were medical students from Hamburg, Germany, who completed the HAM-SJT and HAM-Nat as part of the admission tests between 2019^3^ and 2022 and completed a one-week practical training in general medicine between 2020 and 2023. All participants consented to the research project stav [33] in exchange for detailed feedback on their admission test results. The stav is an association of German faculties with the aim of researching and further developing student selection. The present study used the infrastructure of the stav (e.g., database), but the content of the study was independently designed by the authors. After consenting to stav, participants were asked to respond to an online sociodemographic questionnaire including questions on gender, age, GPA, and professional prequalification.

In 2019, participants in the admission tests were preselected based on their GPA. This year, the HAM-SJT was voluntary and did not count toward the admission decision. From 2020 on, no preselection was done and the HAM-SJT was completed under high-stakes condition as the results were included in two faculty selection quotas responsible for 70% of available study places. Professional prequalification was not included in any admission quota for Hamburg Medical School, but in 2019 there was a quota that considered applicants’ waiting time for admission, indirectly increasing the age and proportion of students with professional prequalification (see Table 1 for a comparison between cohorts from different years). Although the changes in test and admission conditions between 2019 and subsequent years may have resulted in a slightly different composition of students, we decided to include participants from 2019 as the procedures for collecting supervisor ratings did not change, and we assume that most participants completed the HAM-SJT with a high level of conscientiousness since the test was written directly after the relevant HAM-Nat. However, as described in the data analysis section below, we controlled for possible differential relations between cohorts and supervisor ratings.

**Table 1:** Sample Description and Descriptive Statistics per Cohort.


	YEAR OF PRACTICAL TRAINING				

	2020	2021	2022	2023	DIFFERENCE BETWEEN YEARS

*N*	58	29	49	74	

Year of HAM-SJT	2019: 58 (19 from retest)	2020: 29	2021: 43 2020: 6	2022: 56 2021: 17 2020: 1	

SV Assured-Dominant	*M* = 4.92 *SD* = 1.15	*M* = 4.54 *SD* = 1.04	*M* = 4.79 *SD* = 1.25	*M* = 4.42 *SD* = 0.95	*F*(1, 208) = 5.47, ***p* = 0.020**

SV Warm-Agreeable	*M* = 5.86 *SD* = 1.25	*M* = 5.69 *SD* = 1.36	*M* = 6.24 *SD* = 1.37	*M* = 5.77 *SD* = 1.32	*F*(1, 208) = 0.00, *p* = 0.990

SV Emotional Stability	*M* = 5.67 *SD* = 0.89	*M* = 5.53 *SD* = 0.92	*M* = 5.78 *SD* = 1.25	*M* = 5.74 *SD* = 1.00	*F*(1, 208) = 0.40, *p* = 0.528

% Professional Prequal	29	29	22	7	*F*(1, 201) = 10.54, ***p* = 0.001**

HAM-SJT	*M* = 0.45 *SD* = 0.15	*M* = 0.50 *SD* = 0.09	*M* = 0.63 *SD* = 0.12	*M* = 0.59 *SD* = 0.11	*F*(1, 208) = 49.00, ***p* < 0.001**

GPA	*M* = 24.00 *SD* = 5.01	*M* = 25.00 *SD* = 3.18	*M* = 23.70 *SD* = 5.07	*M* = 25.50 *SD* = 2.96	*F*(1, 203) = 2.56, *p* = 0.111

HAM-Nat	*M* = 0.64 *SD* = 0.08	*M* = 0.69 *SD* = 0.11	*M* = 0.69 *SD* = 0.14	*M* = 0.72 *SD* = 0.15	*F*(1, 189) = 8.37, ***p* = 0.004**

% Female	64	55	73	47	*F*(1, 208) = 2.28, *p* = 0.133

Age	*M* = 23.03 *SD* = 3.85	*M* = 21.55 *SD* = 1.92	*M* = 22.33 *SD* = 2.46	*M* = 21.95 *SD* = 2.23	*F*(1, 208) = 3.40, *p* = 0.067


*Note*. Participants could retake the HAM-SJT, and we included the most recent year of HAM-SJT participation, resulting in different numbers in the row “Year of HAM-SJT”. In 2019, *N* = 19 students from the final sample completed the same version of the HAM-SJT from the admission tests as part of a retest during the first week of study. Professional Prequal = percent of participants with completion of healthcare related training before admission. Gender is coded as *0* = male and *1* = female. SV = supervisor rating. GPA = grade point average. Bold p-values are significant at < 0.05.

After the first study year, admitted students completed a mandatory one-week practical training in general practice. During the training, students shadowed a supervising general practitioner and other practice staff in everyday situations to become more familiar with different counselling purposes. They were also required to practice history taking and physical examinations. Students who agreed to take part in this study were rated by a general practitioner and up to two other supervising staff members on different interpersonal dimensions using an adapted paper-pencil version of the validated short form of the Interpersonal Adjective List [34] (see Supplement). To ensure honest ratings, students only received summarized feedback on the cohorts’ performance and no individual feedback. As an incentive, they had the chance to win a 100 euros gift voucher for online shopping.

Supervisors’ ratings were available for 322 students, of whom *N* = 210 had HAM-SJT and sociodemographic data and were included in the final sample (59% female; mean age of 22.28 years at practical training with *SD* = 2.82). Information on professional prequalification was available for *n* = 203 participants of whom 40 had already completed a healthcare related vocational training. On average, participants were evaluated by two raters (range from one to three raters).

### 2.2. Measures

#### 2.2.1. Supervisor Ratings

The rating asked how different adjectives describing either assured-dominant (e.g., confident), warm-agreeable (e.g., considerate), or emotionally stable (e.g., calm) behavior applied to the student on a scale from *1 (extremely inaccurate)* to *8 (extremely accurate)*. We used four adjectives from the short form of the Interpersonal Adjective List [34] for assured-dominant and warm-agreeable behavior, and five adjectives from a research project for emotional stability [35]. Ratings were first averaged across items within each rater and then averaged across raters to obtain one final score per dimension. Internal consistency of the items within each dimension (Cronbach’s alpha) and interrater reliability of the final scores (ICC1k) were good (assured-dominant: 0.71/0.78; warm-agreeable: 0.92/0.77; emotional stability = 0.70/0.66).

#### 2.2.2. Professional Prequalification

We defined professional prequalification as having completed vocational training in a healthcare related profession, which usually lasted at least two years (although there are a few exceptions that lasted less than one year) and included theoretical and practical content. The information was self-reported and coded as *0* if no healthcare related training (including veterinary medicine and pharmacy) was completed and as *1* if healthcare related training was completed.

#### 2.2.3. HAM-SJT

Situations pictured in the SJT include a main character who is studying medicine or dentistry and is facing a challenging interpersonal encounter with fellow students, lecturers, physicians, medical staff, patients, or their relatives. Each situation is followed by three to five behavioral responses that describe possible reactions for the main character. The number of situations and behavioral responses slightly changed between years, but a minimum of 18 situations and 74 behavioral responses were included. Participants were asked to rate the appropriateness of each behavioral response on a scale from *1* (very appropriate) to *4* (very inappropriate). To obtain a final score, participants’ responses were first intraindividually *z*-standardized to control for response styles [36]. In the next step, responses were compared against a panel of subject matter experts by averaging the sum of the squared difference from the intraindividually *z*-standardized expert mean. This score was linearly transformed by subtracting it from the highest occurring score so that a higher score denotes a better performance. When participants took the HAM-SJT more than once, we included the most recent data.

#### 2.2.4. Covariates

Participants’ GPA was self-reported and reflects an aggregate of high school grades across years and exams. The original grade was linearly transformed on a scale from *0 (lowest possible grade)* to *30 (highest possible grade)*. The HAM-Nat included 88 (2019) or 60 (from 2020 on) single-choice natural science questions with five possible solutions. Participants’ answers were coded as *0 (incorrect answer)* or *1 (correct answer)* and averaged across all items to obtain one score reflecting the percent of correct answers. Gender was coded as *0* if participants identified as male and as *1* if identifying as female. Age was computed at the year of practical training.

### 2.3. Statistical Analysis

Data preparation and analysis were performed with R version 4.4.0 [37] and the packages *lme4* and *performance* to analyze mixed models [38,39]. We initially computed Pearson correlations between all variables to determine their zero-order relations. For relations between supervisor ratings and admission criteria (HAM-SJT, GPA, HAM-Nat), we additionally provided correlations controlling for possible range restrictions using Thorndike’s Case 2 [40] and derived the unrestricted standard deviation from the dataset before matching, thus including both admitted and non-admitted participants. Next, we calculated linear mixed models in which supervisor ratings were regressed on covariates only (Step 1), on covariates and professional prequalification (Step 2a) or on covariates and the HAM-SJT (Step 2b).^4^ We included year of practical training as a random effect in each model to account for different relations between cohorts due to changing admission or study conditions. We refer to a significance level of *α* = 0.05 for all analyses and categorize effect sizes of 0.10 as being small, 0.20 as being medium, and 0.30 as being large [41].

## 3. Results

Statistics per year and comparisons are displayed in Table 1, while descriptive statistics and correlations for the whole sample are displayed in Table 2. Cohorts from different years differed significantly in supervisor ratings of assured-dominant behavior (*F*(1, 208) = 5.47, *p* = 0.020), the percent of students with completed vocational training (*F*(1, 201) = 10.54, *p* = 0.001), HAM-SJT performance (*F*(1, 208) = 49.00, *p* < 0.001), and HAM-Nat performance (*F*(1, 189) = 8.37, *p* = 0.004).

**Table 2:** Descriptive Statistics and Correlations.


	*N*	*M*	*SD*	1.	2.	3.	4.	5.	6.	7.	8.

1. SV Assured-Dominant	210	4.66	1.10	–							

2. SV Warm-Agreeable	210	5.90	1.33	**0.38**	–						

3. SV Emotional Stability	210	5.70	1.02	**0.45**	**0.40**	–					

4. Professional Prequalification	203	0.20	0.40	**0.25**	0.08	**0.18**	–				

5. HAM-SJT	210	0.55	0.14	–0.03 (–0.03)	**0.18** (0.20)	0.01 (0.01)	–0.01	–			

6. GPA	205	24.60	4.24	–0.05 (–0.06)	–0.05 (–0.06)	–0.12 (–0.14)	**–0.47**	0.04	–		

7. HAM-Nat	191	0.69	0.13	–0.12 (–0.15)	**–0.15** (–0.19)	–0.01 (–0.01)	–0.01	0.04	**–0.28**	–	

8. Gender	210	0.59	0.49	–0.02	**0.18**	–0.09	–0.03	**0.18**	**0.14**	**–0.18**	–

9. Age	210	22.28	2.82	0.13	0.04	0.12	**0.51**	–0.02	**–0.64**	0.10	–0.09


*Note*. Professional prequalification is coded as *0* = no healthcare related training completed before admission and *1* = healthcare related training completed before admission. Gender is coded as *0* = male and *1* = female. Correlations corrected for range restriction using Thorndike’s Case 2 are shown in parentheses [40]. SV = supervisor rating. GPA = grade point average. Bold coefficients are significant at < 0.05.

There were positive associations between vocational training and ratings of assured-dominant behavior (*r* = 0.25, *p <* 0.001) or emotionally stable behavior (*r* = 0.18, *p* = 0.012), but no significant relations to ratings of warm-agreeable behavior (*r* = 0.08, *p* = 0.262). Participants with vocational training were also more likely to have lower GPA (*r* = –0.47, *p <* 0.001) and an older age (*r* = 0.51, *p <* 0.001). HAM-SJT performance was positively associated with ratings of warm-agreeable ratings (*r* = 0.18, *p* = 0.010), but not significantly related to ratings of assured-dominant behavior (*r* = 0.03, *p* = 0.705) or emotionally stable behavior (*r* = 0.01, *p* = 0.915). When controlling for range restriction in the HAM-SJT due to its relevance for admission, the positive correlation to warm-agreeable ratings slightly increased (*r*_controlled_ = 0.20). Better HAM-SJT performance was associated with female gender (*r* = 0.18, *p* = 0.010).^5^ Warm-agreeable behavior was also associated with female gender (*r* = 0.18, *p* = 0.011) and worse HAM-Nat performance (*r* = –0.15, *p* = 0.041).

Within a random-intercept only model, we quantified the amount of variance in supervisor ratings due to differences between cohorts. However, the assignment to different cohorts accounted for little variance in ratings of warm-agreeable (1%) and assured-dominant (3%) behavior and for no variance in ratings of emotional stability.

The positive relation between professional prequalification and ratings of assured-dominant behavior remained when controlling for all covariates (*β* = 0.21; 95% CI [0.04; 0.39], *p* = 0.019, see Table 3). In other words, those who had already completed a healthcare related vocational training were rated as behaving generally more assured-dominant, beyond differences in GPA, HAM-Nat, gender, and age. When professional prequalification was not included in the model, age significantly predicted ratings of assured-dominant behavior above other covariates and HAM-SJT performance (*β* = 0.21, 95% CI [0.00; 0.42], *p* = 0.047). The significant correlation between professional prequalification and ratings of emotional stability diminished in the regression model when covariates were entered (*β* = 0.09, 95% CI [–0.09; 0.27], *p* = 0.332).

**Table 3:** Summary of Linear Mixed Models with Professional Prequalification or HAM-SJT as Predictor.


PREDICTOR	SV ASSURED-DOMINANT				SV WARM-AGREEABLE				SV EMOTIONAL STABILITY			
		
	*β*	95% CI	*P*	R^2^_GLMM_	*β*	95% CI	*P*	R^2^_GLMM_	*β*	95% CI	*P*	R^2^_GLMM_

Step 1												

GPA	0.02	[–0.20; 0.22]	0.825	0.039	–0.13	[–0.33; 0.07]	0.201	0.070	–0.10	[–0.30; 0.10]	0.345	0.048

HAM-Nat	–0.12	[–0.29; 0.04]	0.145		–0.16	[–0.31; –0.01]	**0.041**		–0.05	[–0.20; 0.10]	0.521	

Gender	–0.07	[–0.21; 0.09]	0.357		0.17	[0.03; 0.32]	**0.023**		–0.10	[–0.24; 0.05]	0.197	

Age	0.22	[0.00; 0.42]	**0.045**		0.10	[–0.10; 0.31]	0.324		0.18	[–0.03; 0.39]	0.088	

Step 2a												

GPA	0.06	[–0.17; 0.26]	0.593	0.066	–0.16	[–0.37; 0.05]	0.144	0.066	–0.07	[–0.28; 0.14]	0.504	0.055

HAM-Nat	–0.11	[–0.28; 0.04]	0.171		–0.15	[–0.31; 0.00]	0.059		–0.04	[–0.20; 0.12]	0.632	

Gender	–0.08	[–0.22; 0.08]	0.305		0.17	[0.02; 0.32]	**0.029**		–0.10	[–0.25; 0.05]	0.185	

Age	0.12	[–0.11; 0.33]	0.292		0.08	[–0.14; 0.30]	0.480		0.16	[–0.06; 0.38]	0.156	

Professional Prequal	0.21	[0.04; 0.39]	**0.019**		–0.02	[–0.20; 0.16]	0.829		0.09	[–0.09; 0.27]	0.332	

Step 2b												

GPA	0.02	[–0.20; 0.22]	0.819	0.039	–0.15	[–0.34; 0.05]	0.146	0.095	–0.10	[–0.30; 0.10]	0.324	0.050

HAM-Nat	–0.12	[–0.29; 0.04]	0.151		–0.18	[–0.33; –0.03]	**0.022**		–0.06	[–0.21; 0.10]	0.482	

Gender	–0.08	[–0.22; 0.09]	0.324		0.13	[–0.01; 0.28]	0.077		–0.11	[–0.26; 0.04]	0.162	

Age	0.21	[0.00; 0.42]	**0.047**		0.08	[–0.12; 0.29]	0.436		0.17	[–0.03; 0.38]	0.103	

HAM-SJT	0.02	[–0.14; 0.18]	0.766		0.17	[0.02; 0.31]	**0.024**		0.05	[–0.10; 0.19]	0.514	


*Note*. Step 1: Model with covariates only. Step 2a: Model with covariates and professional prequalification. Step 2b: Model with covariates and HAM-SJT. Year of practical training was included as random effect in every model but accounted for little variance in the outcome (SV Assured-Dominant: 3%, SV Warm-Agreeable: 1%, SV Emotional Stability: 0%). Professional prequalification is coded as *0* = no healthcare related training completed before admission and *1* = healthcare related training completed before admission. Gender is coded as *0* = male and *1* = female. R^2^_GLMM_ denotes the marginal R^2^ for generalized linear mixed-effects models according to Nakagawa & Schielzeth [42]. SV = supervisor rating. GPA = grade point average. Bold p-values are significant at < 0.05 (computed with Satterthwaite approximation).

Regressions models with the HAM-SJT and all covariates confirmed the positive relationship of HAM-SJT performance (*β* = 0.17, 95% CI [0.02; 0.31], *p* = 0.024) and the simultaneous negative relationship of HAM-Nat performance (*β* = –0.18, 95% CI [–0.33; –0.03], *p* = 0.022) to ratings of warm-agreeable behavior. That is, students who performed better in the HAM-SJT or worse in the HAM-Nat at admission were rated as typically exhibiting warmer and more agreeable behavior, independently of their GPA, gender, or age. When HAM-SJT performance was not included in the model, gender did significantly predict ratings of warm-agreeable behavior above other covariates and professional prequalification (*β* = 0.17, 95% CI [0.02; 0.32], *p* = 0.029).

## 4. Discussion

While previous validity studies on admission criteria assessing personal characteristics often rely on general interpersonal composite scores in high-stakes contexts, we used a behavior-focused approach with supervisor ratings of assured-dominant, warm-agreeable, and emotionally stable behavior after a one-week practical training. The present results demonstrate that students who had completed a healthcare related vocational training before admission to medical school were rated as behaving more assured-dominant and emotionally stable in social interactions. However, when controlling for cognitive admission criteria, gender, and age, the relation to emotionally stable behavior diminished. The resulting effect size for the prediction of assured-dominant behavior by professional prequalification can be categorized as medium and is comparable to the effect sizes reported for the relation between professional prequalification and performance in interviews assessing personal and social skills [11]. Students who performed better on the HAM-SJT at the time of admission were rated as behaving more warm-agreeable, regardless of their cognitive performance and demographic characteristics. The resulting effect size can be classified as medium, being slightly smaller than a pooled coefficient reported in a recent meta-analysis of the general predictive validity of SJTs for medical admission.

Although many undergraduate medical admission processes directly or indirectly consider professional prequalification, the actual benefit for social interactions in everyday clinical work remains uncertain. The present results suggest that experienced students are generally more straightforward, assertive, and assured when performing typical practical tasks with patients in early stages of medical education. These students may have already executed physical examinations or history taking, know how to deal with different patients, and, consequently, appear more confident to supervisors than students who have never interacted with real patients before. Their confidence and experience could be beneficial not only for the trust of patients or colleagues during practical training, but also for faculties to facilitate learning processes (e.g., on interprofessional cooperation; 43). Thus, if medical schools want to admit applicants with a higher level of confidence in social interactions, considering professional prequalification could be a good option. However, all students have access to specific knowledge and experiences through medical studies, and students without professional prequalification may develop the same level of confidence during medical tasks, so that differences are no longer present at graduation [44]. The findings further underline the importance of disentangling effects of professional prequalification from age effects [45]. While the relationship between professional prequalification and emotionally stable behavior partly existed due to general maturity, the level of confident behavior was foremost influenced by previous vocational training and not by age.

The benefit of better performance in the HAM-SJT at admission was evident through more warm and agreeable behavior during the practical training. Considering the time lag and methodological differences between the HAM-SJT as a standardized written test targeting a mixture of skills and supervisor ratings of actual behavior in specific interpersonal dimensions, the significant result is quite remarkable for the construct validity of the HAM-SJT. Even if the HAM-SJT was not specifically designed to assess communal skills of medical applicants, it appears that behaviors such as consideration of others are highly valued by subject matter experts who scored the HAM-SJT. Other studies have revealed a similar unintended focus for traditional SJTs [46,47]. An SJT also has the potential to compensate for negative selection effects of cognitive admission tests on the communal characteristics of applicants as shown by the opposite results of the HAM-SJT and HAM-Nat. Nevertheless, most medical curricula include courses on communication skills through which students develop, for example, their empathetic skills, and initial differences herein may diminish over the course of study [48]. A recent longitudinal study from Switzerland, however, found that personality differences in medical students remain relatively stable over six years of study, with most students increasing their level of agreeableness as an empathy-related trait until graduation [49]. Regardless of the long-term stability of differences in agreeableness, patients who interact with students during their practical training will benefit from more considerate behavior. Therefore, when medical schools want to emphasize communal skills in their undergraduate selection, the use of an SJT seems an appropriate criterion.

### 4.1. Limitations and Future Research

The major strength of the present study is its high external validity, characterized by the close contact between students and supervisors in different actual work situations. However, the disadvantage of high external validity is lower standardization of raters and situations across students. This variability may have led to unreliable differences between students due to different understandings of behavior definitions (e.g., what constitutes calm behavior) and the possibility of observing these behaviors in relevant encounters. One approach to improve standardization in the future is by introducing mandatory rater training or by providing context-specific examples of low and high construct behavior.

We used a selection of interpersonal dimensions that have been previously deemed crucial for physicians and are observable in actual behavior. Based on supervisors’ comments and lower reliability, we suspect that emotional stability was difficult to assess because the construct includes emotional states that must be reported by the persons themselves. This also applies to other personal characteristics that were discussed for experienced applicants, such as motivation [50] or self-reflection [10]. If one is interested in assessing characteristics with a high internal focus, it would be necessary to include students’ self-reports on emotional states and satisfaction.

Our study followed participants for approximately one and a half years, from application to the end of the first year of study. The transition from selection to initial patient contact was crucial to validate how professional prequalification and SJT performance relate to interpersonal behavior independent of communication courses, which mainly start later in medical education. However, a relevant question for the admission processes is what effect communication courses and, more general, medical education have on social skills development and whether applicants need inherent personal characteristics that cannot be taught. Here, a more nuanced distinction between traits (i.e., how someone generally tends to behave) and skills (i.e., how someone is able to behave when the situations requires it; 51) can help to understand how personal characteristics develop. Although both concepts are closely related, skills eventually become more flexible than traits during medical training as students become more familiar with the medical context and situational requirements. Future research on supervisor ratings could explicitly ask how students are able to behave when such behavior is beneficial (e.g., how warm is a student when a patient is desperate). Corresponding studies could also include professional prequalification as a moderator to shed light on the question of why prior healthcare related qualification did not have a systematic effect on students’ warm-agreeable behavior in the present study (potentially, prequalified students are not generally more warm and agreeable but are able to behave this way when the situation requires it), and whether the positive effects on students’ confidence and assertiveness persist or diminish until graduation.

### 4.2. Conclusion

Professional prequalification and SJTs can serve as valuable criteria to screen for personal characteristics in undergraduate medical admission with different foci: while students with prior professional experience were generally more confident in social interactions, students with higher HAM-SJT performance behaved more considerately in social interactions. Medical schools are advised to consider the results when deciding which personal characteristics are required for admission and which can be developed through the course.

## Additional File

The additional file for this article can be found as follows:

10.5334/pme.1571.s1 Supplement. Supervisor rating form, description and results of the construct-driven SJT (CD-SJT).

## Footnotes

We originally planned to analyze the relation between a construct-driven SJT (CD-SJT) and supervisor ratings with correlations and regression analysis and preregistered this intention in July 2020 (http://tiny.cc/6hd7zz). Whereas the HAM-SJT assesses a mixture of social skills, the CD-SJT is explicitly designed to capture participants’ tendencies to respond with agentic or communal behavior when social interactions require such behaviors. However, because the participant rates for supervisor ratings in 2019 and 2020 (when the CD-SJT was administered) were lower than expected, we could not reach the desired statistical power and decided to report the corresponding results in the Supplement.

We are aware that language proficiency may be another factor influencing performance on admission tests, especially on text-based tests such as the HAM-SJT [52]. For the present study, however, we decided against the inclusion of language proficiency as another covariate because the subsample of non-native speakers was quite small (*N* = 26) and native and non-native speakers did not differ in their supervisor ratings (assured-dominant: *t*(28.85) = 0.29, *p* = 0.772; warm-agreeable: *t*(35.01) = –0.06, *p* = 0.954; emotional stability: *t*(32.65) = 0.79, *p* = 0.433).

In 2019, N = 19 students from the final sample completed the same version of the HAM-SJT from the admission tests as part of a retest during the first week of study.

We additionally checked for moderating effects of professional prequalification and HAM-SJT performance by adding an interaction term to regression models with both main effects. However, the interaction term did not reached significance for any kind of supervisor rating.

The performance difference between females and males on the HAM-SJT corresponds to an effect size of *d* = .37. A meta-analysis reported a slightly smaller effect of *d* = .11 for females outperforming males, but with stronger effect sizes for SJTs related to conscientiousness or agreeableness, which may explain the higher effect in the present study [53]. As gender was also related to supervisor ratings of warm-agreeable behavior, we included gender as a covariate in the regression models to control for possible confounding.
