# Supplementary material for: Spoilt for Choice in Undergraduate Medical Admission: Selection of Confident and Considerate Students Using Professional Prequalification and Situational Judgement Test
Source: Perspect Med Educ. 2025 Jun 23;14(1):360–70. doi: 10.5334/pme.1571 (PMC12227088; doi:10.5334/pme.1571)
Supplement: Supplement. — Supervisor rating form, description and results of the construct-driven SJT (CD-SJT). [file pme-14-1-1571-s1.pdf]

**Supplement to:**

# Spoilt for Choice in Undergraduate Medical Admission: Selection of Confident and Considerate Students with Professional Prequalification and Situational Judgement Test

Additional material (i.e., the R code, and a data request) is available via the following link: http://tiny.cc/6hd7zz

**Supervisor Rating Form**

This is a self-generated translation of the relevant parts of the German original rating form. Items are either retrieved from the short form of the Interpersonal Adjective Scale (1) or a research project (2).

**Name of student: last name, first name**

(1) Please assess the extent to which the following statements apply to the student. You can grade your answer between 1 (extremely inapplicable) and 8 (extremely applicable).

Please do not skip any questions, even if some points are more difficult to assess.

**He/she is…**

| | extremely inapplicable | | | | | | | extremely applicable |
|---|---|---|---|---|---|---|---|---|
| | 1 | 2 | 3 | 4 | 5 | 6 | 7 | 8 |
| ...calm. | ☐ | ☐ | ☐ | ☐ | ☐ | ☐ | ☐ | ☐ |
| ...direct. | ☐ | ☐ | ☐ | ☐ | ☐ | ☐ | ☐ | ☐ |
| ...cordial. | ☐ | ☐ | ☐ | ☐ | ☐ | ☐ | ☐ | ☐ |
| ...anxious. | ☐ | ☐ | ☐ | ☐ | ☐ | ☐ | ☐ | ☐ |
| ...dominant. | ☐ | ☐ | ☐ | ☐ | ☐ | ☐ | ☐ | ☐ |
| ...empathetic. | ☐ | ☐ | ☐ | ☐ | ☐ | ☐ | ☐ | ☐ |
| ...stress-resistant. | ☐ | ☐ | ☐ | ☐ | ☐ | ☐ | ☐ | ☐ |
| ...assertive. | ☐ | ☐ | ☐ | ☐ | ☐ | ☐ | ☐ | ☐ |
| ...harmonious. | ☐ | ☐ | ☐ | ☐ | ☐ | ☐ | ☐ | ☐ |
| ...nervous. | ☐ | ☐ | ☐ | ☐ | ☐ | ☐ | ☐ | ☐ |
| ...confident. | ☐ | ☐ | ☐ | ☐ | ☐ | ☐ | ☐ | ☐ |
| ...considerate. | ☐ | ☐ | ☐ | ☐ | ☐ | ☐ | ☐ | ☐ |
| ...anxious. | ☐ | ☐ | ☐ | ☐ | ☐ | ☐ | ☐ | ☐ |

(2) Open comments (e.g. comments on the questionnaire, further information on the student)

**Thank you for your evaluation!**

**Description of Construct-Driven Situational Judgement Test**

The construct-driven situational judgement test (CD-SJT) was developed in accordance with guidelines for the construct-driven approach (3) and a detailed description can be retrieved from the validation paper by Mielke et al. (4). Both SJTs, the HAM-SJT and the CD-SJT, were implemented in the admission tests for Hamburg Medical School under low-stakes condition in 2019 and under high-stakes condition in 2020. However, the CD-SJT did not influence the admission decision in 2020.

The CD-SJT included an agency scale and a communion scale that covered similar behaviors as the corresponding supervisor ratings of agency (i.e., assure-dominant behavior) and communion (i.e., warm-agreeable behavior). Each scale was assessed with 15 scenarios which were written from a first-person perspective and described a social encounter of a medical student followed by three possible behavioral responses that denoted different levels of agency or communal behavior. Every scenario also included a triggering circumstance to activate agency or communion and a dilemma that argued against highly agentic or communal behavior. Example triggers are interactions with supervisors who do not commit to previous promises or who assigned inappropriate or repetitive tasks (agency) and desperate, help-seeking, or helpless patients or fellow students (communion). Participants were asked to select that behavioral response which aligns most to their behavior in that situation. The scenarios slightly changed between 2019 and 2020 but the assessed constructs and the number of items remained. In 2020, a subsample of $n = 19$ received only ten scenarios with the instruction to rank all three behavioral responses and we used the first ranked behavioral response as their final answer. Participants' answers were than recoded according to their construct level and a final score per scale was computed by averaging across all

corresponding items. If participants completed the CD-SJT in 2019 and 2020, we used their more recent score from 2020.

**Table S1** Descriptive Statistics and Correlations

| | *N* | *M* | *SD* | 1. | 2. | 3. | 4. | 5. | 6. | 7. | 8. |
|---|---|---|---|---|---|---|---|---|---|---|---|
| 1. SV Assured-Dominant | 108 | 4.77 | 1.13 | - | | | | | | | |
| 2. SV Warm-Agreeable | 108 | 5.98 | 1.26 | 0.53* | - | | | | | | |
| 3. SV Emotional Stability | 108 | 5.63 | 0.99 | 0.38* | 0.52* | - | | | | | |
| 4. CD-SJT Agency | 108 | 2.20 | 0.27 | 0.06 | 0.00 | 0.04 | - | | | | |
| 5. CD-SJT Communion | 108 | 2.55 | 0.23 | 0.07 | 0.12 | -0.01 | -0.32* | - | | | |
| 6. GPA | 108 | 24.00 | 4.30 | 0.05 | -0.03 | -0.08 | 0.17 | -0.05 | - | | |
| 7. HAM-Nat | 91 | 0.68 | 0.11 | -0.17 | -0.19 | 0.01 | -0.11 | 0.12 | -0.41* | - | |
| 8. Gender | 108 | 0.61 | 0.49 | 0.03 | 0.20* | -0.06 | 0.18 | 0.15 | 0.19* | -0.20 | - |
| 9. Age | 108 | 22.69 | 3.18 | 0.04 | 0.01 | 0.10 | -0.08 | -0.07 | -0.72* | 0.22* | -0.18 |

*Note*. Gender is coded as *0* = male and *1* = female. SV = supervisor rating. CD-SJT = construct-driven SJT. GPA = grade point average. *$p$ at least < 0.05

**Table S2** Sample Description and Descriptive Statistics per Cohort

| | Year of Practical Training | | | | |
|---|---|---|---|---|---|
| | 2020 | 2021 | 2022 | 2023 | Difference between years |
| *N* | 54 | 29 | 16 | 9 | |
| Year of CD-SJT | 2019: 54 | 2020: 29 | 2020: 15 | 2020: 9 | |
| | (34 from retest) | | 2019: 1 | | |
| CD-SJT Agency | $M = 2.32$ | $M = 2.06$ | $M = 2.13$ | $M = 2.11$ | $F(1, 106) = 12.76, p < 0.001$ |
| | $SD = 0.19$ | $SD = 0.29$ | $SD = 0.35$ | $SD = 0.12$ | |
| CD-SJT Communion | $M = 2.43$ | $M = 2.67$ | $M = 2.71$ | $M = 2.56$ | $F(1, 106) = 18.22, p < 0.001$ |
| | $SD = 0.19$ | $SD = 0.20$ | $SD = 0.20$ | $SD = 0.26$ | |

*Note.* CD-SJT = construct-driven SJT.

**Table S3** Summary of Linear Mixed Models With CD-SJT Agency or Communion as Predictor

| | SV Assured-Dominant | | | | SV Warm-Agreeable | | | | SV Emotional Stability | | | |
|---|---|---|---|---|---|---|---|---|---|---|---|---|
| Predictor | $\beta$ | 95% CI | $p$ | $R^2_{GLMM}$ | $\beta$ | 95% CI | $p$ | $R^2_{GLMM}$ | $\beta$ | 95% CI | $p$ | $R^2_{GLMM}$ |
| Step 1 | | | | | | | | | | | | |
| GPA | 0.06 | [-0.31; 0.43] | 0.743 | 0.046 | -0.25 | [-0.60; 0.10] | 0.171 | 0.111 | -0.09 | [-0.44; 0.26] | 0.630 | 0.044 |
| HAM-Nat | -0.19 | [-0.42; 0.04] | 0.105 | | -0.24 | [-0.46; -0.03] | 0.029* | | -0.06 | [-0.28; 0.16] | 0.596 | |
| Gender | -0.02 | [-0.24; 0.19] | 0.824 | | 0.19 | [-0.01; 0.39] | 0.073 | | -0.07 | [-0.27; 0.13] | 0.497 | |
| Age | 0.22 | [-0.13; 0.57] | 0.221 | | 0.15 | [-0.18; 0.48] | 0.392 | | 0.22 | [-0.11; 0.56] | 0.197 | |
| Step 2a | | | | | | | | | | | | |
| GPA | 0.05 | [-0.32; 0.43] | 0.791 | 0.047 | -0.25 | [-0.61; 0.10] | 0.169 | 0.110 | -0.11 | [-0.47; 0.24] | 0.544 | 0.054 |
| HAM-Nat | -0.19 | [-0.42; 0.04] | 0.108 | | -0.24 | [-0.46; -0.03] | 0.030* | | -0.06 | [-0.27; 0.16] | 0.601 | |
| Gender | -0.03 | [-0.25; 0.18] | 0.770 | | 0.18 | [-0.02; 0.39] | 0.084 | | -0.09 | [-0.29; 0.11] | 0.403 | |
| Age | 0.22 | [-0.13; 0.57] | 0.221 | | 0.15 | [-0.18; 0.48] | 0.393 | | 0.23 | [-0.11; 0.56] | 0.193 | |
| CD-SJT Agency | 0.05 | [-0.15; 0.25] | 0.647 | | 0.02 | [-0.17; 0.21] | 0.852 | | 0.10 | [-0.09; 0.29] | 0.308 | |
| Step 2b | | | | | | | | | | | | |
| GPA | 0.09 | [-0.28; 0.46] | 0.627 | 0.068 | -0.22 | [-0.58; 0.12] | 0.228 | 0.114 | -0.08 | [-0.44; 0.27] | 0.658 | 0.044 |
| HAM-Nat | -0.20 | [-0.43; 0.02] | 0.084 | | -0.25 | [-0.47; -0.04] | 0.025* | | -0.06 | [-0.28; 0.15] | 0.579 | |
| Gender | -0.05 | [-0.26; 0.16] | 0.629 | | 0.16 | [-0.03; 0.37] | 0.127 | | -0.08 | [-0.28; 0.13] | 0.471 | |
| Age | 0.22 | [-0.13; 0.57] | 0.222 | | 0.14 | [-0.18; 0.48] | 0.420 | | 0.22 | [-0.11; 0.56] | 0.201 | |
| CD-SJT Communion | 0.15 | [-0.07; 0.35] | 0.183 | | 0.10 | [-0.11; 0.30] | 0.341 | | 0.03 | [-0.17; 0.23] | 0.749 | |

*Note*. Year of practical training was included as random effect but accounted for little variance in the outcome (SV Assured-Dominant: 0%, SV Warm-Agreeable: 2%, SV Emotional Stability: 0%). Gender is coded as *0* = male and *1* = female. $R^2_{GLMM}$ denotes the marginal $R^2$ for generalized linear mixed-effects models according to Nakagawa & Schielzeth (5). SV = supervisor rating. GPA = grade point average.* $p < 0.05$ (computed with Satterthwaite approximation)

1. Jacobs I, Scholl W. IAL-K: Entwicklung einer Kurzform der Interpersonalen Adjektivliste. Diagnostica. 2016;62(4):227-41.
2. Niemeyer L, Back MB, Nestler S, Ryvkina E. Exact 2021 [Available from: https://osf.io/67m4y/.
3. Guenole N, Chernyshenko OS, Weekly J. On designing construct driven situational judgment tests: Some preliminary recommendations. Int J Test. 2017;17(3):234-52.
4. Mielke I, Breil SM, Amelung D, Espe L, Knorr M. Assessing distinguishable social skills in medical admission: does construct-driven development solve validity issues of situational judgment tests? BMC Med Educ. 2022;22:Article 293.
5. Nakagawa S, Schielzeth H. A general and simple method for obtaining R2 from generalized linear mixed-effects models. Methods Ecol Evol. 2013;4(2):133-42.
